# 2,4-Dichloro­pyrimidine

**DOI:** 10.1107/S1600536809019667

**Published:** 2009-05-29

**Authors:** Yan Chen, Zheng Fang, Ping Wei

**Affiliations:** a College of Biotechnology and Pharmaceutical Engineering, Nanjing University of Technolgy, Xinmofan Road No. 5 Nanjing, Nanjing 210009, People’s Republic of China; b School of Pharmaceutical Sciences, Nanjing University of Technolgy, Xinmofan Road No. 5 Nanjing, Nanjing 210009, People’s Republic of China

## Abstract

The mol­ecule of the title compound, C_4_H_2_Cl_2_N_2_, is almost planar [maximum deviation = 0.013 (3) Å for a Cl atom]. In the crystal structure, inter­molecular C—H⋯N inter­actions link the mol­ecules into chains.

## Related literature

For a related structure, see: Bhasin *et al.* (2009 ▶). For bond-length data, see: Allen *et al.* (1987 ▶).
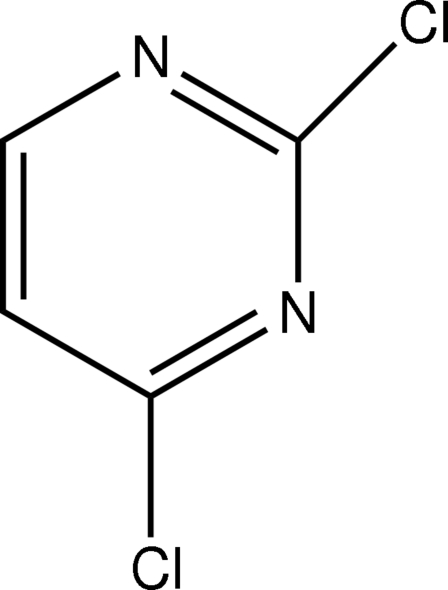


## Experimental

### 

#### Crystal data

C_4_H_2_Cl_2_N_2_
*M* *_r_* = 148.98
Monoclinic, 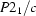

*a* = 7.5090 (15) Å
*b* = 10.776 (2) Å
*c* = 7.1980 (14) Å
β = 92.92 (3)°
*V* = 581.7 (2) Å^3^
*Z* = 4
Mo *K*α radiation
μ = 0.99 mm^−1^
*T* = 294 K
0.30 × 0.20 × 0.20 mm

#### Data collection

Enraf–Nonius CAD-4 diffractometer
Absorption correction: ψ scan (North *et al.*, 1968 ▶) *T*_min_ = 0.755, *T*_max_ = 0.826
1223 measured reflections
1139 independent reflections
733 reflections with *I* > 2σ(*I*)
*R*_int_ = 0.084
3 standard reflections frequency: 120 min intensity decay: 1%

#### Refinement

*R*[*F* ^2^ > 2σ(*F* ^2^)] = 0.069
*wR*(*F* ^2^) = 0.180
*S* = 1.01
1139 reflections
73 parameters
H-atom parameters constrained
Δρ_max_ = 0.39 e Å^−3^
Δρ_min_ = −0.32 e Å^−3^

### 

Data collection: *CAD-4 Software* (Enraf–Nonius, 1989 ▶); cell refinement: *CAD-4 Software*; data reduction: *XCAD4* (Harms & Wocadlo, 1995 ▶); program(s) used to solve structure: *SHELXS97* (Sheldrick, 2008 ▶); program(s) used to refine structure: *SHELXL97* (Sheldrick, 2008 ▶); molecular graphics: *ORTEP-3 for Windows* (Farrugia, 1997 ▶) and *PLATON* (Spek, 2009 ▶); software used to prepare material for publication: *SHELXL97*.

## Supplementary Material

Crystal structure: contains datablocks global, I. DOI: 10.1107/S1600536809019667/hk2698sup1.cif

Structure factors: contains datablocks I. DOI: 10.1107/S1600536809019667/hk2698Isup2.hkl

Additional supplementary materials:  crystallographic information; 3D view; checkCIF report

## Figures and Tables

**Table 1:** Hydrogen-bond geometry (Å, °)

*D*—H⋯*A*	*D*—H	H⋯*A*	*D*⋯*A*	*D*—H⋯*A*
C1—H1*B*⋯N2^i^	0.93	2.62	3.548 (7)	174

Symmetry code: (i) 
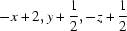
.

**Fig. 1.**
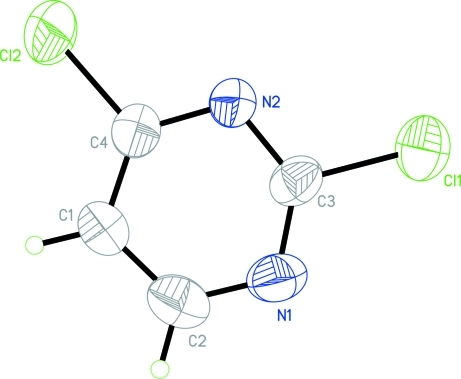
The molecular structure of the title molecule, with the atom-numbering scheme. Displacement ellipsoids at the 30% probability level.

**Fig. 2.**
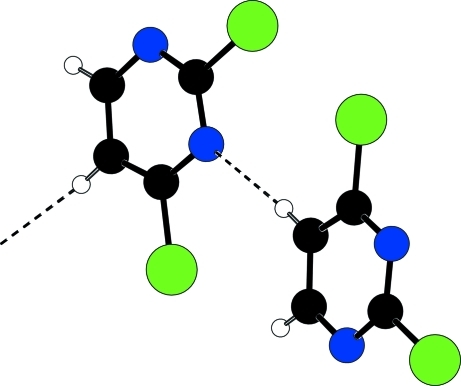
A partial packing diagram of the title compound. Hydrogen bonds are shown as dashed lines.

## supplementary crystallographic information

### Comment

Some derivatives of pyrimidine are important chemical materials. We report herein the crystal structure of the title compound.

In the molecule of the title compound (Fig 1), the bond lengths (Allen *et al.*,  1987) and angles are within normal ranges. Ring A (N1/N2/C1-C4) is, of course, planar. Atoms Cl1 and Cl2 are 0.012 (3) and 0.013 (3) Å away from the ring plane, respectively. So, the molecule is planar.

In the crystal structure, intermolecular C-H···N interactions (Table 1) link the molecules into chains (Fig. 2), in which they may be effective in the stabilization of the structure.

### Experimental

For the preparation of the title compound, uracil (100 g, 0.82 mol) was dissolved in phosphorous oxychloride (400 ml) in a two-necked round-bottom flask (500 ml) equipped with a condenser. The solution was refluxed with stirring for 3.5 h at 383 K. The residual phosphorous oxychloride was removed in vacuo at 323 K, and the remaining oil was poured into ice (50 g) followed by extraction with chloroform (3 × 50 ml). The combined organic extract was washed with dilute sodium carbonate solution and dried over anhydrous sodium sulfate. The title compound was obtained by evaporation of solvent (Bhasin *et al.*, 2009). Crystals suitable for X-ray analysis were obtained by slow evaporation of a methanol solution.

### Refinement

H atoms were positioned geometrically, with C-H = 0.93 Å for aromatic H and constrained to ride on their parent atoms, with U_iso_(H) = 1.2U_eq_(C).

### Crystal data

**Table:**

C_4_H_2_Cl_2_N_2_	*F*(000) = 296
*M**_r_* = 148.98	*D*_x_ = 1.701 Mg m^−^^3^
Monoclinic, *P*2_1_/*c*	Mo *K*α radiation, λ = 0.71073 Å
Hall symbol: -P 2ybc	Cell parameters from 25 reflections
*a* = 7.5090 (15) Å	θ = 10–13°
*b* = 10.776 (2) Å	µ = 0.99 mm^−^^1^
*c* = 7.1980 (14) Å	*T* = 294 K
β = 92.92 (3)°	Block, colorless
*V* = 581.7 (2) Å^3^	0.30 × 0.20 × 0.20 mm
*Z* = 4	

### Data collection

**Table:**

Enraf–Nonius CAD-4 diffractometer	733 reflections with *I* > 2σ(*I*)
Radiation source: fine-focus sealed tube	*R*_int_ = 0.084
graphite	θ_max_ = 26.0°, θ_min_ = 2.7°
ω/2θ scans	*h* = −9→0
Absorption correction: ψ scan (North *et al.*, 1968)	*k* = 0→13
*T*_min_ = 0.755, *T*_max_ = 0.826	*l* = −8→8
1223 measured reflections	3 standard reflections every 120 min
1139 independent reflections	intensity decay: 1%

### Refinement

**Table:**

Refinement on *F*^2^	Primary atom site location: structure-invariant direct methods
Least-squares matrix: full	Secondary atom site location: difference Fourier map
*R*[*F*^2^ > 2σ(*F*^2^)] = 0.069	Hydrogen site location: inferred from neighbouring sites
*wR*(*F*^2^) = 0.180	H-atom parameters constrained
*S* = 1.01	*w* = 1/[σ^2^(*F*_o_^2^) + (0.07*P*)^2^ + 1.45*P*] where *P* = (*F*_o_^2^ + 2*F*_c_^2^)/3
1139 reflections	(Δ/σ)_max_ < 0.001
73 parameters	Δρ_max_ = 0.39 e Å^−^^3^
0 restraints	Δρ_min_ = −0.32 e Å^−^^3^

### Special details

**Table:**

Geometry. All e.s.d.'s (except the e.s.d. in the dihedral angle between two l.s. planes) are estimated using the full covariance matrix. The cell e.s.d.'s are taken into account individually in the estimation of e.s.d.'s in distances, angles and torsion angles; correlations between e.s.d.'s in cell parameters are only used when they are defined by crystal symmetry. An approximate (isotropic) treatment of cell e.s.d.'s is used for estimating e.s.d.'s involving l.s. planes.
Refinement. Refinement of *F*^2^ against ALL reflections. The weighted *R*-factor *wR* and goodness of fit *S* are based on *F*^2^, conventional *R*-factors *R* are based on *F*, with *F* set to zero for negative *F*^2^. The threshold expression of *F*^2^ > σ(*F*^2^) is used only for calculating *R*-factors(gt) *etc*. and is not relevant to the choice of reflections for refinement. *R*-factors based on *F*^2^ are statistically about twice as large as those based on *F*, and *R*- factors based on ALL data will be even larger.

### Fractional atomic coordinates and isotropic or equivalent isotropic displacement parameters (Å^2^)

**Table:**

	*x*	*y*	*z*	*U*_iso_*/*U*_eq_	
Cl1	0.5768 (2)	0.63809 (14)	0.0975 (2)	0.0762 (6)	
Cl2	1.1679 (2)	0.83010 (16)	0.3510 (3)	0.0862 (7)	
N1	0.6273 (6)	0.8744 (4)	0.0912 (7)	0.0612 (12)	
N2	0.8628 (5)	0.7492 (4)	0.2211 (6)	0.0555 (11)	
C1	0.8975 (7)	0.9681 (5)	0.2072 (8)	0.0652 (15)	
H1B	0.9665	1.0384	0.2321	0.078*	
C2	0.7272 (8)	0.9751 (5)	0.1252 (9)	0.0689 (16)	
H2B	0.6808	1.0526	0.0927	0.083*	
C3	0.7027 (6)	0.7699 (5)	0.1403 (7)	0.0485 (12)	
C4	0.9577 (7)	0.8520 (5)	0.2490 (8)	0.0562 (14)	

### Atomic displacement parameters (Å^2^)

**Table:**

	*U*^11^	*U*^22^	*U*^33^	*U*^12^	*U*^13^	*U*^23^
Cl1	0.0693 (9)	0.0670 (10)	0.0897 (12)	−0.0184 (7)	−0.0222 (8)	−0.0065 (8)
Cl2	0.0581 (9)	0.0731 (11)	0.1233 (15)	−0.0074 (7)	−0.0370 (9)	−0.0038 (10)
N1	0.055 (3)	0.059 (3)	0.068 (3)	0.013 (2)	−0.009 (2)	0.002 (2)
N2	0.052 (2)	0.042 (2)	0.071 (3)	−0.0014 (18)	−0.017 (2)	−0.002 (2)
C1	0.070 (4)	0.042 (3)	0.081 (4)	0.000 (2)	−0.013 (3)	−0.007 (3)
C2	0.079 (4)	0.047 (3)	0.081 (4)	0.006 (3)	0.000 (3)	0.007 (3)
C3	0.045 (2)	0.056 (3)	0.044 (3)	0.005 (2)	−0.006 (2)	−0.007 (2)
C4	0.051 (3)	0.052 (3)	0.064 (3)	−0.002 (2)	−0.008 (3)	−0.005 (3)

### Geometric parameters (Å, °)

**Table:**

Cl1—C3	1.725 (5)	N2—C4	1.327 (6)
Cl2—C4	1.723 (5)	C1—C2	1.383 (8)
N1—C2	1.334 (7)	C1—C4	1.358 (7)
N1—C3	1.302 (6)	C1—H1B	0.9300
N2—C3	1.327 (6)	C2—H2B	0.9300
			
C3—N1—C2	114.9 (5)	C1—C2—H2B	118.9
C4—N2—C3	113.2 (4)	N1—C3—N2	129.5 (5)
C4—C1—C2	115.8 (5)	N1—C3—Cl1	115.9 (4)
C4—C1—H1B	122.1	N2—C3—Cl1	114.6 (4)
C2—C1—H1B	122.1	N2—C4—C1	124.4 (5)
N1—C2—C1	122.2 (5)	N2—C4—Cl2	115.0 (4)
N1—C2—H2B	118.9	C1—C4—Cl2	120.5 (4)
			
C3—N1—C2—C1	−0.2 (9)	C4—N2—C3—Cl1	178.8 (4)
C4—C1—C2—N1	0.7 (10)	C3—N2—C4—C1	2.5 (9)
C2—N1—C3—N2	1.0 (9)	C3—N2—C4—Cl2	−179.2 (4)
C2—N1—C3—Cl1	−179.9 (4)	C2—C1—C4—N2	−2.0 (10)
C4—N2—C3—N1	−2.0 (9)	C2—C1—C4—Cl2	179.8 (5)

### Hydrogen-bond geometry (Å, °)

**Table:**

*D*—H···*A*	*D*—H	H···*A*	*D*···*A*	*D*—H···*A*
C1—H1B···N2^i^	0.93	2.62	3.548 (7)	174

Symmetry codes: (i) −*x*+2, *y*+1/2, −*z*+1/2.
